# Comparative genomics of chemosensory protein genes (CSPs) in twenty-two mosquito species (Diptera: Culicidae): Identification, characterization, and evolution

**DOI:** 10.1371/journal.pone.0190412

**Published:** 2018-01-05

**Authors:** Ting Mei, Wen-Bo Fu, Bo Li, Zheng-Bo He, Bin Chen

**Affiliations:** Chongqing Key Laboratory of Vector Insects; Chongqing Key Laboratory of Animal Biology; Institute of Entomology and Molecular Biology, Chongqing Normal University, Chongqing, P.R. China; USDA Agricultural Research Service, UNITED STATES

## Abstract

Chemosensory proteins (CSP) are soluble carrier proteins that may function in odorant reception in insects. CSPs have not been thoroughly studied at whole-genome level, despite the availability of insect genomes. Here, we identified/reidentified 283 CSP genes in the genomes of 22 mosquitoes. All 283 CSP genes possess a highly conserved OS-D domain. We comprehensively analyzed these CSP genes and determined their conserved domains, structure, genomic distribution, phylogeny, and evolutionary patterns. We found an average of seven CSP genes in each of 19 *Anopheles* genomes, 27 CSP genes in *Cx*. *quinquefasciatus*, 43 in *Ae*. *aegypti*, and 83 in *Ae*. *albopictus*. The *Anopheles* CSP genes had a simple genomic organization with a relatively consistent gene distribution, while most of the Culicinae CSP genes were distributed in clusters on the scaffolds. Our phylogenetic analysis clustered the CSPs into two major groups: CSP1-8 and CSE1-3. The CSP1-8 groups were all monophyletic with good bootstrap support. The CSE1-3 groups were an expansion of the CSP family of genes specific to the three Culicinae species. The Ka/Ks ratios indicated that the CSP genes had been subject to purifying selection with relatively slow evolution. Our results provide a comprehensive framework for the study of the CSP gene family in these 22 mosquito species, laying a foundation for future work on CSP function in the detection of chemical cues in the surrounding environment.

## Introduction

Over time, insects have developed a complete chemosensing system to perceive chemical cues from external environment [1]. Discerned olfactory stimuli are conveyed into the central nervous system via electrical signal transduction, producing a series of behavioral responses such as feeding, courtship, and avoidance to adapt to the external environment [1,2]. The lymph of the insect sensillum houses all processes and interactions by which the environmental odorant molecules reach the nerve membrane receptors [1]. Several classes of olfactory proteins have been reported to be involved in chemosensory perception, including odorant-binding proteins (OBPs), chemosensory proteins (CSPs), odorant receptors (ORs), ionotropic receptors (IRs), and sensory neuron membrane proteins (SNMPs) [3]. Generally, OBPs and CSPs represent two functionally similar classes of carrier proteins, dissolving and transporting chemical signals or other stimuli of lipophilic compounds to the chemosensory receptors. CSPs are major binding proteins in insects, and they are primarily differentiated from OBPs in that they bind and carry non-volatile odorants and semiochemicals [4,5]. The earliest identified members of the CSP family, isolated from the antennae of *Drosophila melanogaster*, were the olfactory specific protein D (OS-D), the OS-D-like protein [6,7], and the pheromone-binding protein A-10 (A-10) [8]. Subsequently identified CSPs include CLP-1 [9], p10 [10], the sensory appendage protein (SAP) [7,11,12], and the CSPs themselves [13,14]. While these proteins have been early described in relation with the insect olfactory system [13], there is no solid evidence to support what remains an odd assumption. An increasing number of studies are rather in strong agreement with a role of CSPs in immune responses not only in moths, but also in flies and whiteflies [15–18]. This is also supported by ubiquitous tissue distribution and ontogeny in expression of CSP genes, such as the labial palp, maxilla, pheromone gland, wing, and leg [5,13,19]. CSPs are all small, globular, soluble proteins that possess a conserved cysteine CSP motif (C1-X_6-8_-C2-X_16-21_-C3-X_2_-C4) containing two disulfide bonds (C1-X_6-8_-C2, C3-X_2_-C4) [20,21]. The disulfide bonds in the CSP motif are inter-helical with two small loops, forming a rigid hydrophobic pocket involved in ligand binding [22–24]. All CSPs shuttle in the aqueous lymphatic fluid of chemosensilla [1]. Some CSPs are involved in chemosensory signal transduction, in the solubilization of pheromone components [5,14]. Other CSPs affect the physiological processes and behavior of insects, e.g., moulting [25], tissue formation or regeneration [10,15,26], reproduction [27], and resistance reactions [28].

Recently, CSPs have been identified in insect species besides *D*. *melanogaster*, including *Camponotus japonicas* [29], *Heliothis virescens* [30], *Apis mellifera* [31], *Bombyx mori* [32], and *Anopheles gambiae* [33]. Forêt *et al*. (2007) analyzed the members of the CSP gene family in *Ap*. *mellifera* using both bioinformatic annotation and expression profiling, and compared these CSPs with those in other arthropods to better understand their evolution and function [31]. Pelletier and Leal (2011) identified different families of olfactory proteins (OBPs, CSPs, SNMPs) in *Culex quinquefasciatus*, and analyzed the characterization and expression of genes in these three families [34]. Kulmuni *et al*. (2013) identified and annotated the CSP genes in the genomes of seven ant species, and studied their evolution [35]. Vieira and Rozas (2011) conducted an exhaustive comparative genomic analysis of the CSP and OBP gene families in 20 Arthropoda species, giving insight into the origin and evolutionary history of these two gene families [36]. Neafsey *et al*. (2015) sequenced and assembled the genomes and transcriptomes of 16 anopheline mosquitoes [37]. However, the genes in the CSP family have not been identified in these mosquito species, nor have they been thoroughly analyzed at the whole-genome level.

Mosquitoes are regarded as the deadliest animals to humans due to their capacity for infectious disease transmission [38]. At present, 22 mosquito genomes are available, providing a foundation for a comparative genomic study of mosquito CSPs. Here, we identified and annotated the CSP genes of these 22 mosquito species at the whole-genome level, and analyzed the characteristics and conserved domains of these CSPs using bioinformatic techniques. We constructed a phylogeny to inform mosquito classification. We also determined the rate of sequence evolution (non-synonymous to synonymous changes, Ka/Ks), and the putative orthologous CSPs across the mosquito species. Finally, we investigated species-specific expansions in the Culicinae subfamily using comparative genomic methods. This work provides a framework for study of the CSP family of genes in mosquitoes, and provides a basis for further study of the specific role of CSPs in the detection of chemical signals in the surrounding environment by insects.

## Materials and methods

### Genome sequence sources

We downloaded 21 previously published assembled genome and transcriptome mosquito from VectorBase (https://www.vectorbase.org) [37,39–51]. We obtained the annotated genome (unpublished) and transcriptome of an additional species, *An*. *sinensis*, from the Institute of Entomology and Molecular Biology of Chongqing Normal University, China (see Table 1 for details of all data stated above, [52]). We also downloaded four CSP gene sequences from *D*. *melanogaster* (GenBank accession ID: CAG26928, NP_001286809, NP_001286871, and AAF49381) from GenBank (http://www.ncbi.nlm.nih.gov/).

**Table 1:** Source of genomes used in the study and numbers of putative CSPs found in each genome.

Subfamily Genus/Subgenus/Series	Species	Abbreviations of species	Genome version	Genome assembly ID^a^	Genome size (Mb)	Putative CSPs	Genome assembly reference	Transcriptome assembly reference
Anophelinae						130		
*Anopheles/Nyssorhynchus*	*An*. *darlingi*	Ada	AdarC3	GCA_000211455.3	173.92	4	Marinotti *et al*. (2013)	unpublished
	*An*. *albimanus*	Aal	AalbS1	GCA_000349125.1	165.33	8	Neafsey *et al*. (2015)	Neafsey *et al*. (2015)
*Anopheles/Anopheles*	*An*. *sinensis*	Asi	unpublished	unpublished	327.22	8	unpublished	Chen *et al*. (2014)
	*An*. *atroparvus*	Aat	AatrE1	GCA_000473505.1	217.57	7	Neafsey *et al*. (2015)	Neafsey *et al*. (2015)
*Anopheles/Cellia/*Neomyzomyia	*An*. *farauti*	Afa	AfarF2	GCA_000473445.2	175.52	8	Neafsey *et al*. (2015)	Neafsey *et al*. (2015)
	*An*. *dirus*	Adi	AdirW1	GCA_000349145.1	209.79	8	Neafsey *et al*. (2015)	Neafsey *et al*. (2015)
*Anopheles/Cellia/*Myzomyia	*An*. *funestus*	Afu	AfunF1	GCA_000349085.1	218.45	8	Neafsey *et al*. (2015)	Crawford *et al* (2010)
	*An*. *minimus*	Ami	AminM1	GCA_000349025.1	195.70	6	Neafsey *et al*. (2015)	Neafsey *et al*. (2015)
	*An*. *culicifacies*	Acu	AculA1	GCA_000473375.1	198.03	7	Neafsey *et al*. (2015)	Neafsey *et al*. (2015)
*Anopheles/Cellia/*Neocellia	*An*. *maculatus*	Ama	AmacM1	GCA_000473185.1	141.20	6	Neafsey *et al*. (2015)	Neafsey *et al*. (2015)
	*An*. *stephensi*	Ast	AsteS1	GCA_000349045.1	216.26	6	Jiang *et al*. (2014)	Gokhale *et al*. (2013)
*Anopheles/Cellia/*Pyretophorus	*An*. *epiroticus*	Aep	AepiE1	GCA_000349105.1	216.83	7	Neafsey *et al*. (2015)	Neafsey *et al*. (2015)
	*An*. *christyi*	Ach	AchrA1	GCA_000349165.1	169.04	6	Neafsey *et al*. (2015)	Neafsey *et al*. (2015)
	*An*. *gambiae*	Aga	AgamP4/ AgamS1	GCA_000005575.2 GCA_000150785.1	268.44/ 229.28	8	Holt *et al*.(2002) Lawniczak *et al*. (2010)	Neafsey *et al*. (2015)
	*An*. *arabiensis*	Aar	AaraD1	GCA_000349185.1	239.13	7	Neafsey *et al*. (2015)	Neafsey *et al*. (2015)
	*An*. *quadriannulatus*	Aqu	AquaS1	GCA_000349065.1	275.35	6	Neafsey *et al*. (2015)	Neafsey *et al*. (2015)
	*An*. *merus*	Amr	AmerM2	GCA_000473845.2	244.34	7	Neafsey *et al*. (2015)	Neafsey *et al*. (2015)
	*An*. *melas*	Aml	AmelC2	GCA_000473525.2	222.01	6	Neafsey *et al*. (2015)	Neafsey *et al*. (2015)
	*An*. *coluzzii*	Aco	AcolM1	GCA_000150765.1	218.22	7	Lawniczak *et al*. (2010)	Cassone *et al*. (2014)
Culicinae						153		
*Culex*	*Cx*. *quinquefasciatus*	Cqu	CpipJ2	GCA_000209185.1	574.57	27	Arensburger *et al*. (2010)	Lv *et al*. (2016)
*Aedes*	*Ae*. *aegypti*	Aae	AaegL3	GCA_000004015.1	1342.21	43	Nene *et al*. (2007)	Vogel *et al*. (2017)
	*Ae*. *albopictus*	Aao	AaloF1	GCA_001444175.1	1868.07	83	Chen *et al*. (2015)	Esquivel *et al*. (2016)

^a^ All assembled mosquito genomes and transcriptomes were downloaded from VectorBase (https://www.vectorbase.org), except for those of *An*. *sinensis*. The genome and transcriptome of *An*. *sinensis* were sequenced and annotated by the Chongqing Normal University with the genome data not yes published, and the transcriptome sequences published in Chen *et al*. (2014).

### Genome-wide identification of CSPs

To find all putative CSPs in the 22 mosquito species, we first performed several rounds of exhaustive BlastP respectively searches against the amino acid (aa) databases of these mosquitoes using characterized CSP sequences from GenBank (http://www.ncbi.nlm.nih.gov/) as queries (with an E-value threshold of 10^−5^). Second, we built Hidden Markov Model (HMM) profiles downloaded from the Pfam database (http://pfam.xfam.org/) [53] using OS-D (for CSP; Pfam ID: PF03392), and performed an HMM search against the aa database of each mosquito species. Third, we performed a tBlastN search against each the genome assembly of each mosquito species using the corresponding aa sequences obtained in the previous two steps (with an E-value threshold of 10^−5^). Using this procedure, we repeatedly identified candidate CSPs until no new hits were found. We manually removed duplicate sequences. We obtained the raw nucleotide sequences of all candidate CSPs directly from the genome. Finally, we used Fgenesh (http://www.softberry.com) [54] to predict the candidate CSP genes, and then confirmed all candidate CSP genes against CSP conserved domain information (OS-D, Pfam03392) with SMART (http://smart.embl-heidelberg.de/) [55]. We only considered sequences belonging to the OS-D superfamily as putative CSPs. All CSP genes were verified by BlastN searches against the EST database of each species (with E-value threshold of 1x10^-15^). All putative CSP genes were classified and named according to their characteristics and phylogenetic relationships.

### Analysis of CSP characteristics

For each predicted aa sequence encoded by our putative CSP genes, we calculated the molecular weight and the theoretical isoelectric point (pI) using ExPASy ProtParam (http://web.expasy.org/protparam/). We predicted the subcellular location with TargetP (v1.1; http://www.cbs.dtu.dk/services/TargetP/) [56]; the N-terminus signal peptides with PrediSi (http://www.predisi.de) [57]; and the secondary structure with PSIPRED (v3.3; http://bioinf.cs.ucl.ac.uk/psipred) [58]. We observed the structures of the putative CSP genes, including the intron phase, with GSDS (http://gsds.cbi.pku.edu.cn/index.php) [59], based on the corresponding coding and the genomic sequences of the putative CSP genes obtained.

The multiple alignments of CSP aa sequences of the 22 mosquito species were conducted by ClustalX [60], and the alignment parameters were set to the defaults with gap-opening and gap extension penalties 10.0 and 0.2, respectively. Each group of CSP aa sequences (CSP1-8 and CSE1-8, established based on the phylogeny below and previously published works) were further separated, aligned, and displayed with Genedoc [61]. The conserved domains of the CSP aa sequences were identified through the NCBI conserved domain database (CDD) with default setting (http://www.ncbi.nlm.nih.gov/Structure/cdd/wrpsb.cgi)We checked our multiple alignments against these conserved domains. We interrogated the occurrence frequencies of each CSP aa over the 22 mosquito species, displayed with WebLogo 3 (http://weblogo.threeplusone.com/).

To locate the CSP genes within each of the 22 mosquito genomes, we performed tBlastN search against current assembly of each genome sequence of 22 mosquito species using the corresponding aa sequence of CSP gene. We considered genes located within 20Kb of each other a gene cluster [62]. We drew plots displaying the genomic distribution of the CSP genes on the chromosomes of the 22 mosquitos, as well as scaffolds and contigs manually with Photoshop CS8. We compared the distributions of CSP genes across a subgroup of 19 *Anopheles* species.

### Phylogenetics and evolution analysis

The best-fit models (JTT) produced by using automatically generated trees in MEGA5 [63] for the putative CSP aa sequences of the 22 mosquitos and *D*. *melanogaster*, and we used maximum likelihood (ML) to construct unrooted phylogenies of these sequences with MEGA5 [63]. Bootstrap values were calculated with 1000 replicates; bootstrap values ≥ 50% were marked on the branches of the unrooted ML trees constructed. We used the Interactive Tree of Life (http://itol.embl.de/) to display the trees [64].

To investigate selection pressures on the CSP genes, we calculated the Ka/Ks ratio (non-synonymous (Ka) to synonymous (Ks) substitution rates) for the gene group of CSP1-8 and CSE1-3, as well as all CSP genes for each mosquito species. We aligned the sequences, and constructed the tree topology of each CSP group with MEGA5 using default parameters. The consensus sequence of each gene group was used as reference in the Ka/Ks ratio calculation. We compared different substitution models in HyPhy [65] and in PAML (the program codeml) [66,67], and estimated Ka/Ks for each CSP group using the most conservative model chosen using HyPhy and PAML.

## Results and discussion

### Identification of CSP genes and divergence across mosquito species

**Fig 1:**
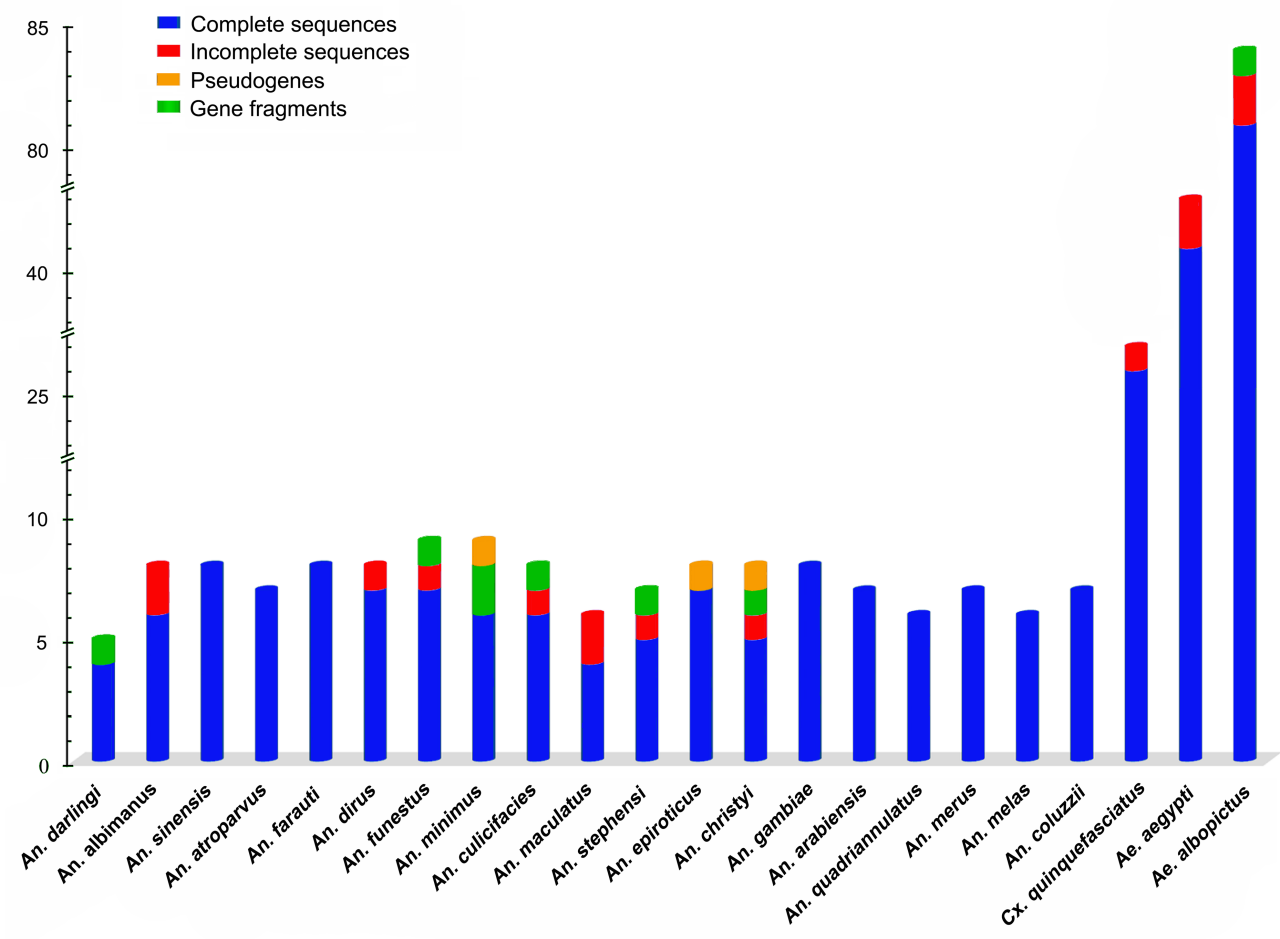
Numbers of CSP genes in the 22 mosquito species investigated. Gene numbers include complete, incomplete, and gene fragments as well as pseudogenes. Genes with complete and incomplete sequences are considered putatively functional CSP genes. Gene fragments sequences and pseudogenes are not used in further analysis.

We identified a total of 130 putative CSP genes from the genomes of these 19 *Anopheles* species, with an average of seven CSP genes per species (4 CSP genes in 1 species; 6 in 6 species; 7 in 6 species, and 8 in 6 species). We identified 153 putative CSP genes from the genomes of the three Culicinae species: 27 in *Cx*. *quinquefasciatus*, 43 in *Aedes aegypti*, and 83 in *Ae*. *albopictus* (Table 1, Fig 1). Among the 283 CSP genes identified across the 22 mosquito species, 269 genes are complete protein-coding sequences extracted from the genome assemblies. The remaining 14 genes (*AalCSP4*, *AalCSP5*, *AchCSP5*, *AcuCSP5*, *AdiCSP5*, *AfuCSP5*, *AmaCSP4*, *AmaCSP5*, *AstCSP5*, *CquCSP27*, *AaeCSP3*, *AaeCSP37*, *AaoCSP23* and *AaoCSP67*) were not complete due to incomplete genomic sequences. Out of the 283 genes, 266 were supported by transcriptome data but 17 not (*AcoCSP8*, *AepCSP5*, *AfaCSP7*, *CquCSP1*, *AaeCSP1*, and 12 *AaoCSPs* for *Ae*. *albopictus*, see S1 File for details). The aa sequences corresponding to these 269 genes all had four conserved cysteines as well as a CSP family domain (OS-D). We therefore considered all 269 genes as members of CSP family (i.e. insect pheromone-binding family or A10/OS-D, Pfam number: PF03392). An additional eight short gene fragments (coding length <54 aa) with high similarities to CSPs, and three pseudogenes (AmiCSP6, AepCSP4, AchCSP6) lacking the characteristic CSP domain were not used in any analysis (Fig 1).

In the 22 mosquito species investigated, the CSP number of each species ranged from four genes in *An*. *darling* to 83 genes in *Ae*. *albopictus*. Based on the greater number of CSP genes found in the Culicinae species, we suggest that the CSP family expanded largely in the subfamily Culicinae mosquitoes in terms of the gene numbers. Various numbers of CSP genes have been reported in other insect species: one CSP gene is known in *Thricolepisma aurea* and *Folsomia candida* [68]; six are known in *Pediculus humanus* [36] and *Ap*. *mellifera* [31]; 13 are known in *Acyrthosiphon pisum* [69]; 20 are known in *Tribolium castaneum* [70] and *B*. *mori* [5]; and 70 are known in *Locusta migratoria* [71]. The gene numbers could be also divergent within the same genus or family: the number of CSP genes varies from 3 to 4 across *Drosophila* [36] and from 11 to 21 among ants [35]. CSP genes have also been identified in very limited number of non-insect arthropods, although the number of CSPs is much less on average: only one CSP gene has been found *Ixodes scapularis* and *Artemia franciscana*; two in *Archispirostreptus gigas* and *Triops cancriformis*; and three in *Daphnia pulex* [36,68]. All of these results showed that the numbers of the CSP family of genes were highly variable and their evolution was divergent and dynamic among species [36,68]. CSP genes might be subject to different evolutionary pressures in different species through gene lost or gain, based on the need for different molecular mechanisms with which to detect and identify complex odor components. It is likely that some insects, such as *Ae*. *albopictus* (83 CSP genes) and *L*. *migratoria* (70 CSP genes), might require large numbers of different carrier proteins for chemosensing.

### Characterization of identified CSP genes

Across the 130 CSPs of the 19 *Anopheles* species, the theoretical isoelectric points (pI) ranged from 4.5 to 10.4; the aa sequence length ranged from 68 to 181 aa; and the molecular weight (Mw) ranged 8.2 to 20.2 kDa. We do not include CSP5s in these ranges as it had an unusually long aa sequence (187–331 aa) due to C-terminal extension, and a consequently high Mw (21.1–36.4 kDa). Across the 153 CSPs of the three Culicinae species, the pI ranged from 4.4 to 10.4; the aa sequence length ranged from 71 to 25; and the Mw ranged from 7.7 to 26.9. The CSPs of these two groups of mosquitoes were generally comparable. Most of these 283 CSPs (82.3%) encoded secretory pathway proteins with a 14 to 33 aa N-terminus signal peptide. The remaining CSP genes lacked a signal peptide: six genes in the shared CSP group (*AfaCSP7*, *CquCSP1*, *AaoCSP3*, *AfuCSP5*, *AmaCSP5* and *AaeCSP3*, although the sequences of the last three might be incomplete) and all 40 AaeCSPs and AaoCSPs genes in CSE3 group (the Culicinae-specific expansion CSP groups) (see phylogenetic analysis section). The subcellular location showed that two genes (*AfaCSP7* and *AaeCSP3*) belonged to mitochondria targeting peptides. Analysis of the predicted secondary structure of the CSPs suggested that almost all full-length CSPs were characterized by six α-helix structures (S1 Fig).

**Fig 2:**
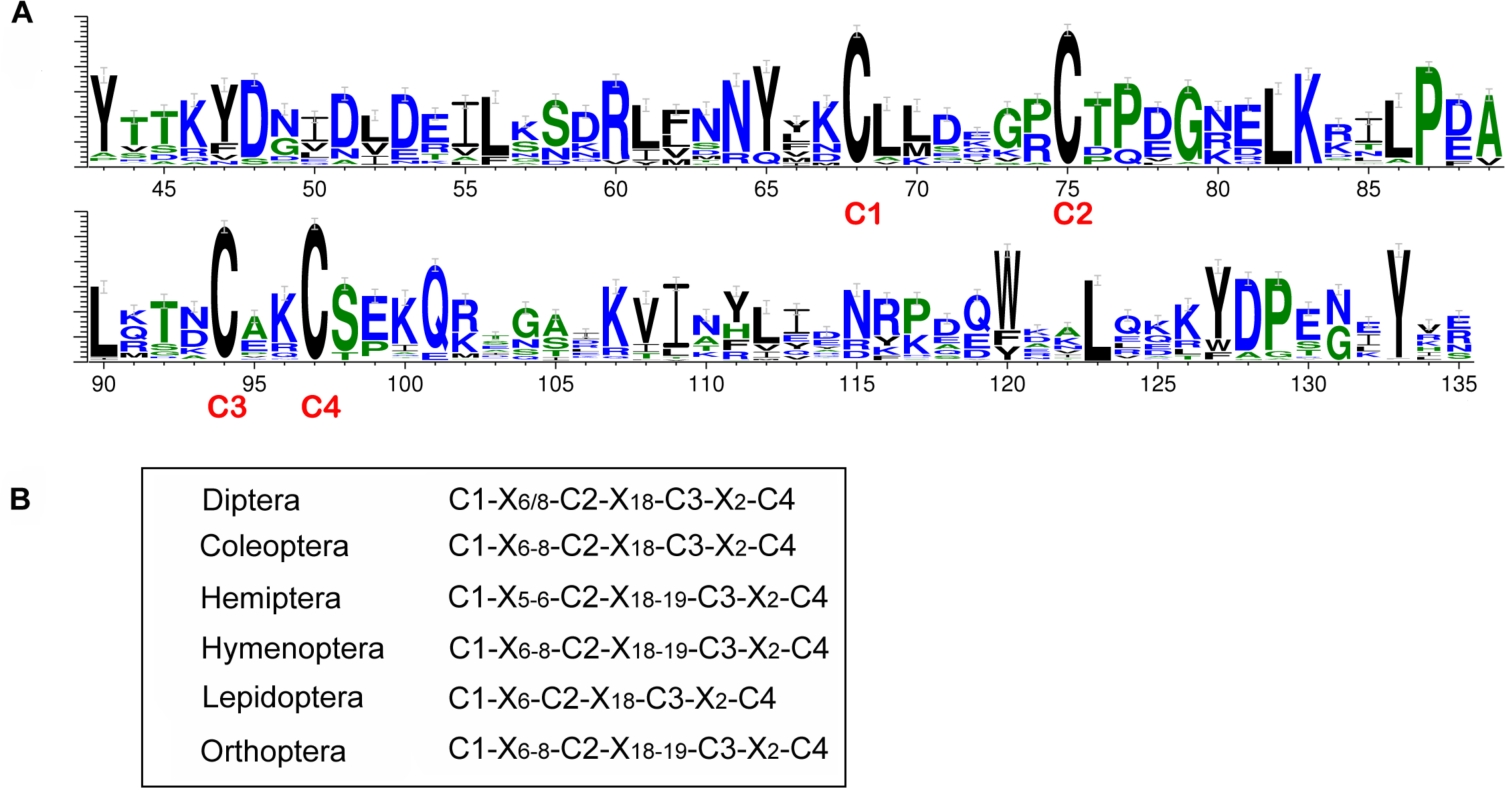
Conserved amino acid sequences and domains of the CSP family. A) The conserved aa sequences with frequency of aa occurrence for the 22 mosquito genomes. B) The conserved four cysteines domain (C1-C4) in different orders of insects. X_6/8_ indicates 6 or 8 non-cysteine aa; X_18_ is 18 aa; X_6-8_ is 6–8 aa.

We investigated the frequency of conserved regions based on the alignment of CSPs across all species tested. A total of 269 CSPs (95.1% of the total CSPs) had complete conserved OS-D domain sequences (Pfam motif: Pfam03392). Of these, 243 had a 93 aa domain, while in an additional 26 the domain length ranged from 75 aa to 125 aa. The remaining 14 CSPs (4.9%) had a conserved OS-D domain but lacked at least one aa due to sequence incompleteness (see phylogenetic analysis section). Within the OS-D domain of complete sequences, four cysteine residues were completely conserved with two disulfide bonds linking each pair of neighboring cysteines (C1-X_6_-C2, C3-X_2_-C4) (Fig 2A). This pattern of conserved cysteines (C-Pattern, C1-X_6_-C2-X_18_-C3-X_2_-C4) was comparable to those identified in other groups of insects (Fig 2B). Such high conservation across all mosquito species examined means that these genes carry out critical conserved functions. The insect CSP C-Pattern was: the first and second cysteines were separated by 5 to 8 non-cysteine aa residues; the third and fourth cysteines were separated by two non-cysteine aa residues; and the second and third cysteines were separated by 18 or 19 non-cysteine aa residues [72]. This C-Pattern might be different in non-insects: there were 12 and 1 or 3 residues between the first and second pair of cysteines in the *Julida* genus of millipedes [33].

### Structure, location, and expansion of CSP genes

The eight groups of CSPs shared by the 22 mosquito species all had relatively consistent genomic structures (S1 File). Sequences belonging to the CSP1, CSP2, and CSP3 groups had a single exon from 381 bp to 390 bp long; the CSP4, CSP6, and CSP7 sequences had two exons; the CSP8 sequences had two or three exons; and the CSP5 sequences had four or five exons. Four CSP sequences did not follow this pattern (*AmaCSP5*, *AfuCSP5*, *AcuCSP5*, and *AaeCSP3*), but these might have been incomplete. Both the CSP5 and CSP7 groups differed with respect to the number and size of exons between the *Anopheles* and the Culicinae species. Three of the CSE groups of genes (CSE1, CSE2, and CSE3) had only one exon, except for two AaoCSPs (*AaoCSP14* and *AaoCSP44*) that had two exons. All 283 genes had 0 to 4 introns varying widely in size (from 29 bp to 24,165 bp). Of these, eight had introns longer than 10, 000 bp (*AarCSP7*, *AgaCSP7*, *AsiCSP5*, *AsiCSP7*, *CquCSP2*, *AaeCSP2*, *AaeCSP37* and *AaoCSP2*). All characteristics of the 283 CSP genes are listed in S1 File.

**Fig 3:**
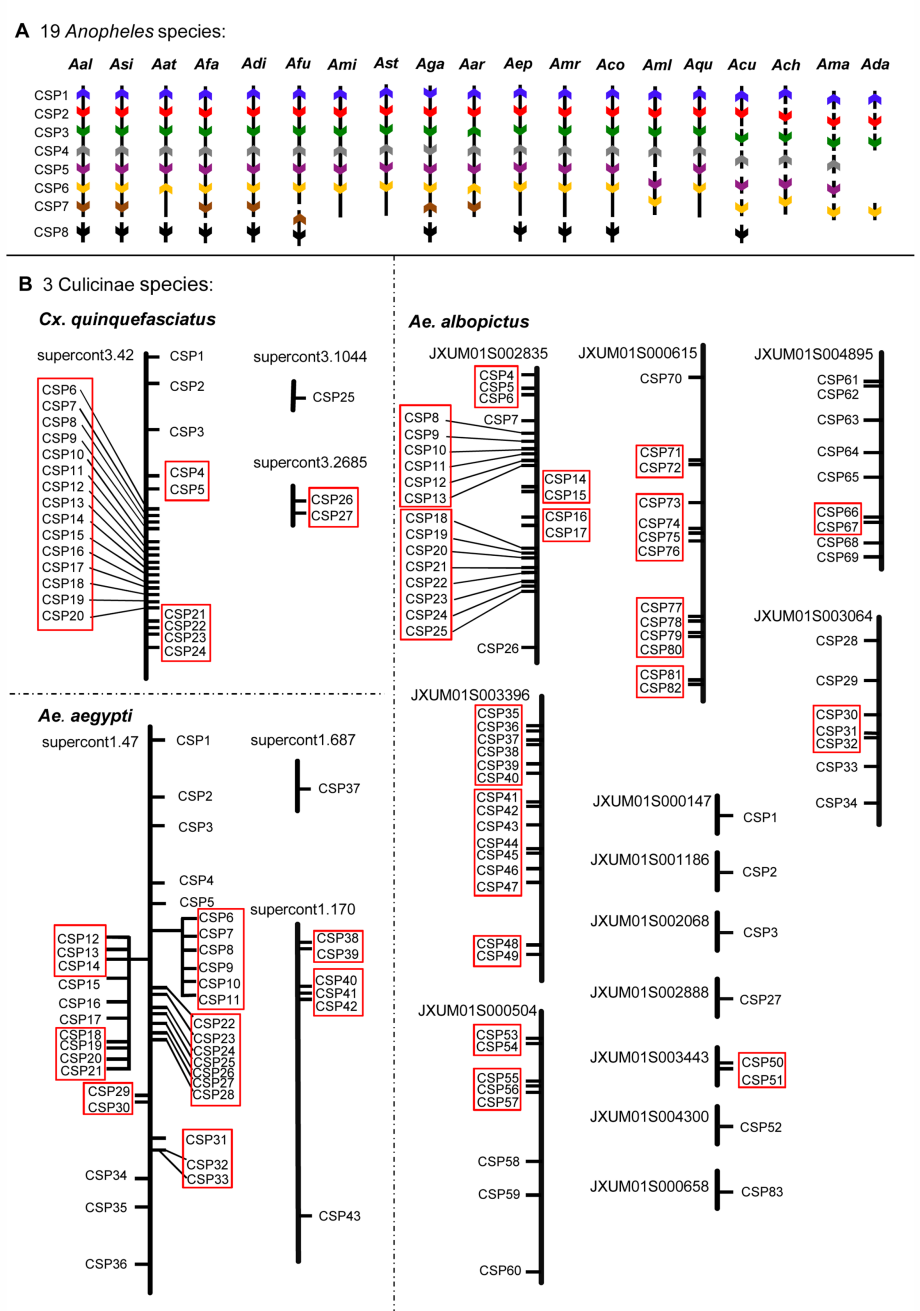
Genomic localization of CSP genes in 22 mosquito species. A) Relative positions of CSP genes in the 19 *Anopheles* species. The eight horizontal bars indicate eight different genes, marked in different colors with arrows showing the 5’-3’ direction of the sequences. B) Location of CSP genes in the three Culicinae species, with the 27–83 genes of each species marked in relative genomic distance. Gene clusters are represented by a red box.

We located the CSP genes of each of the 22 mosquito species on the corresponding genomes (Fig 3). In 18 of *Anopheles* species, groups CSP1-6 are distributed in 1 to 6 scaffolds with the same relative positions. The exception is *An*. *darlingi* (Ada), which lacks CSP4 and CSP5 possibly due to genomic sequence incompleteness. All of the genes in groups CSP1-6 have the same gene direction except for *AgaCSP1*, *AarCSP3*, *AgaCSP4*, *AatCSP6*, and *AarCSP6* (Fig 3A). Only seven species (Aal, Asi, Afa, Adi, Afu, Aga and Aar) have the CSP7 gene. All CSP7 genes are adjacent to CSP6 in a single scaffold (except for *AfuCSP7* that is in another scaffold) and all have the same gene direction (except for *AfuCSP7* and *AgaCSP7*). Eleven species have the CSP8 gene, all with same gene direction, but located in separate scaffolds. These results suggest that the genomic organization of CSP1-7 in *Anopheles* is relatively simple, with these CSPs located close together, while CSP8 is rather more distant. Compared with the CSP genes of *B*. *mori*, *D*. *melanogaster*, *Ap*. *mellifera* and *T*. *castaneum*, the genomic organization shows clear genetic differentiation between different species [7,17,18]. For instance, all *BmorCSP* genes (20 *BmorCSP*, except *BmorCSP10*, *13* and *19*) sit close to each other in the same genomic region that span over 104337 bps separated in average by about 2000–20000 bps [18]. Four *DmelCSP* genes, two of them (*DmelAAM68292* and *DmelPhk3*) are located within 5 Kb of each other, and approximately 900 Kb from *DmelPebIII* on chromosome 2R, the rest of the *DmelCSP* gene (*Dmelos-d*) is located on chromosome 3L [7].

In *Cx*. *quinquefasciatus*, the 27 *CquCSP*s were located on three different scaffolds (supercont3.42, 3.1044, and 3.2685, supercont indicating some large contig). Most of these (89%; *CquCSP1*-*24*) were distributed in the same scaffolds of supercont3.42, with *CquCSP4-24* gathered into a large clusters (Fig 3B). In *Ae*. *aegypti*, 83.7% of the CSPs (36 of 43) were located in supercont1.47; 14.0% in supercont1.170 (6 of 43) and 2.3% in supercont1.687 (1 of 43; *AaeCSP37*). In this species, 30 CSP genes were gathered into eight clusters. In *Ae*. *albopictus*, the 75 of the 83 CSP genes were located on 13 different scaffolds: 23 in JXUM01S002835, 15 in JXUM01S003396, 13 in JXUM01S000615, 9 in JXUM01S004895, 8 in JXUM01S000504, and seven JXUM01S003064. The remaining eight CSPs were located in seven different contigs. Of the 83 CSP genes, 24 genes only have 10 different aa sequences, That is, *AaoCSP8*, *AaoCSP10*, *AaoCSP11*, and *AaoCSP39* with identical aa sequences, and *AaoCSP9*, *AaoCSP12*, *AaoCSP37*, and *AaoCSP40* as well. In contrast to the *Anopheles* species, most of CSP genes of the three Culicinae species were distributed in clusters on the scaffolds, and were obviously expanded, likely through a series of gene duplication events. This was particularly noticeable in *Ae*. *albopictus*, where the CSP genes seemed to be the most expanded, and those genes with same aa sequences might experience most recent gene duplications through transposition of transcripts.

### Phylogenetics of CSP genes in the 22 mosquito species

**Fig 4:**
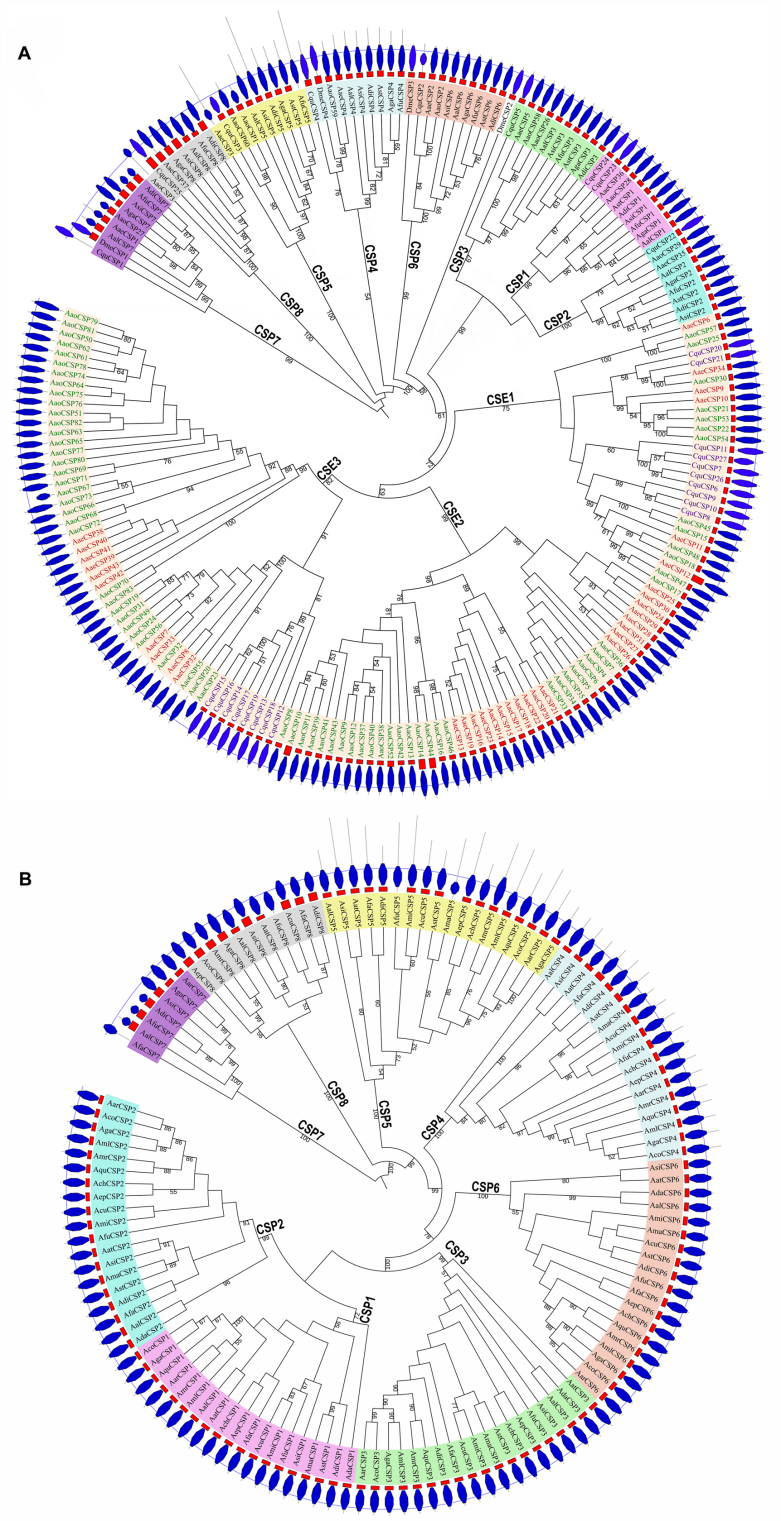
Phylogeny of the CSP aa sequences of 22 mosquito species and *D*. *melanogaster*. A) Phylogeny of CSP genes in six representative *Anopheles* species, three Culicinae species, and *D*. *melanogaster*. B) Phylogeny of CSP genes in all 19 *Anopheles* species. ML phylogenetic trees were constructed using the WAG (Whelan and Goldman) model, as selected by ModTest. Bootstrap values were calculated using 1000 replications. Bootstrap values ≥50% are marked on branches. The most outed layer of blue octagon denotes the position and size of the domain OS-D. The second outed layer of red rectangles shows the signal peptide and its relative size. CSP: Shared CSP groups; CSE: Culicinae-specific expanded CSP groups. In the CSE groups, CquCSPs are shown in purple, AaeCSPs in red, AaoCSPs in green. In the CSP groups, *Anopheles* CSPs are shown in black.

We constructed two unrooted ML trees based on the CSP aa sequences, using the best-fit model of evolution (WAG) as selected by ModTest. One ML tree (the "partial" tree) included six representative *Anopheles* species, three Culicinae species, and *D*. *melanogaster* (Fig 4A). The other ML tree included the 19 *Anopheles* species (Fig 4B). In the former tree, CSP genes were clearly separated into 11 groups with high bootstrap support (≥ 67%): the shared groups CSP1-8 in all mosquitoes and the CSE1-3 for Culicinae-specific expansion groups (CSE being abbreviated for the Culicinae-specific expansion CSP groups). We named CSP1-8 based on conventions used in previous studies [31,34,70]. The names of CSE1-3 are novel, as earlier naming conventions were neither unified nor meaning-specific (e.g. OS-D in *D*. *melanogaster* [6] and *L*. *migratoria* [73]; and SAP in *Manduca sexta* [74] and *An*. *gambiae* species [11]). In the CSP1, CSP3, and CSP5 groups of *Cx*. *quinquefasciatus* and *Ae*. *albopictus*, three sister pairs of CSP genes might each have experienced a single gene duplication event (*CquCSP23* and *CquCSP24; AaoCSP26* and *AaoCSP58*; and *AaoCSP1* and *AaoCSP60*). Three CSPs of *D*. *melanogaster* (*DmeCSP4*, *DmeCSP3*, and *DmeCSP1*) grouped into CSP4, CSP6, and CSP7 groups, respectively, suggesting that the origin of these CSP groups predates the divergence of the mosquito and *Drosophila* lineages. The CSP5 and CSP8 groups have no homologs in *D*. *melanogaster*, suggesting that these groups originated after the split of mosquito and *Drosophila* lineages; alternatively, the homologs of *D*. *melanogaster* may have been lost. In addition, our results suggest that *DmeCSP2* showed to be a sister group with complexes of other groups (i.e. groups CSP1-3 and groups CSE1-3), suggesting that these groups may have expanded in mosquitoes in order to adapt to environmental change during evolution. There are 18 *CquCSP*s, 35 *AaeCSP*s, and 73 *AaoCSP*s in the groups CSE1-3. In the three Culicinae species, there are many more CSPs in these groups than there are in the groups CSP1-8. The CSE1 and CSE3 gene groups are present in all three Culicinae species investigated, but the CSE2 group was missing in *Cx*. *quinquefasciatu*s, suggesting that CSE1-3 did not originate for all Culicinae mosquitoes.

In the CSP phylogenetic tree for all 19 *Anopheles* species (Fig 4B), the 130 CSP genes were also classified into eight groups (CSP1-8) with high bootstrap support (≥ 99%; except for CSP1 with only 72% support). In each of the groups CSP1, CSP2, CSP3, and CSP6, we found a single CSP gene in each of the 19 *Anopheles* species, suggesting that these groups of genes are conserved in *Anopheles*, and that each group of genes may have a similar chemosensing function. In *An*. *gambiae*, it has been suggested that four groups (*AgaCSP1*, *AgaCSP2*, *AgaCSP3*, and *AgaCSP6*, corresponding to *AgaSAP1*, *AgaSAP2*, *AgaSAP3*, and *AgaCSP1*) have independently evolved a common function, these groups are phylogenetically close to honeybee CSP3, a protein known to be involved in the binding of brood pheromone components [33]. The CSP4 and CSP5 genes are present in all *Anopheles* species except for *An*. *darling*. In *An*. *gambiae*, *AgaCSP4* (as *AgaCSP3*) showed a completely different spectrum of binding, and suggested to play a more specific role [33]. The function of *AgaCSP5* was unknown. Only eight of the *Anopheles* species investigated have CSP7 genes, and only 13 have CSP8. *AgaCSP7* (as *AgaCSP4*) and *AgaCSP8* (as *AgaCSP6*) in *An*. *gambiae* are phylogenetically close to the honeybee gene *AmelCSP5*, which encodes a protein previously reported to be involved in embryo development [33].

Amino acid sequence identities was high within the groups CSP1 (65–100%,), CSP2 (70–100%), CSP3 (75–100%), CSP4 (45–99%), CSP6 (73–100%), and CSP8 (74–98%; S1 Fig, S2 File). The CSP7 group was present in only seven *Anopheles* species and three Culicinae species; aa sequence identities among those species was 33–100% slightly lower than those in the CSP1-CSP4, CSP6 and CSP8 groups (S2 File). Overall, aa sequence identities within the CSP5 group was low (19–99%), although we found higher aa sequence identities among the *Anopheles* species alone (57–99%), suggesting that there is great variation in CSP5 between *Anopheles* species and Culicinae species. Sequence identities among the three Culicinae species was higher (CSE1, 56–100%; CSE2, 77–100%; and CSE3, 56–100%), and the groups were more conserved (S2 File, S2 Fig).

Two major families of proteins in the chemosensory system, OBPs and CSPs, were thought to belong to a superfamily of general binding proteins, and to share a common ancestor near the origin of the arthropods. We searched the sequences of these two families in GenBank to investigate their origins [36]. We found CSPs in the arthropod subphylums Hexapoda, Crustacea, Myriapoda, and Chelicerata; CSPs were especially widespread in the insects. In contrast, OBPs were only present in the subphylum Hexapoda. These results suggest that CSPs originated earlier than OBPs, consistent with other studies [36,68,75,76]. We also found fewer, more variable CSP genes as compared to OBP genes, again consistent with previous work [36,68]. We were unable to construct a well-supported phylogenetic tree of all known CSP genes to confidently show their phylogenetic relationship. Finally, we only investigated the phylogenetic relationship of CSPs of mosquito species in this study.

### Evolution of CSP genes

The comparison tests of three different substitution models (M0 (one-ratio), Branch model and Site model) using the program codeml in PAML and three models (SLAC, FEL and REL) in HyPhy with default parameters showed that the M0 and SLAC (single likelihood ancestor counting) were more conservative than other two, respectively. Therefore, the M0 and SLAC were applied in the subsequent analyses with the program codeml and HyPhy, respectively. The Ka/Ks ratios for CSP1-8 for all 22 mosquito species tested varied from 0.08 to 0.38 when estimated with HyPhy, and from 0.09 to 0.36 when estimated with PAML. The Ka/Ks ratios for CSE1-3 for the three Culicinae species ranged from 0.07 to 0.16 when estimated with HyPhy, and from 0.07 to 0.17 when estimated with PAML (Table 2). The Ka/Ks ratios of all CSP genes as a whole for 22 mosquito species ranged from 0.19 to 0.47 when estimated with HyPhy, and from 0.13 to 0.53 when estimated with PAML. The average Ka/Ks ratio across all mosquito species was 0.29 (HyPhy) and 0.30 (PAML) (S3 File). The rates of Ka/Ks change are important for the understanding of the environmental selection pressures on genes in the dynamics species evolution [77,78]. Purifying selection is indicated when the value of Ka/Ks < 1, neutral evolution when Ka/Ks = 1, and positive selection when Ka/Ks > 1 [66]. Generally, as the Ka/Ks ratio decreases, the tolerated selection pressure of the gene increases; small Ka/Ks ratios indicate a highly conserved gene [79]. The largest Ka/Ks ratio we calculated was 0.53, suggesting that these CSP genes are likely subject to purifying selection with relatively slow evolution and high conservation. The results are consistent with orthologous groups of CSP genes in ant species, which are also highly conserved and thought to be under purifying selection [35].

**Table 2:** Ka/Ks ratios of each group of CSP genes in the 22 mosquito species, as calculated by HyPhy and PAML.

	HyPhy Ka/Ks	PAML Ka/Ks
Shared CSP groups		
CSP1	0.15	0.23
CSP2	0.09	0.11
CSP3	0.12	0.19
CSP4	0.30	0.31
CSP5	0.38	0.36
CSP6	0.08	0.09
CSP7	0.34	0.28
CSP8	0.24	0.23
Culicinae-specific expansion CSP groups		
CSE1	0.16	0.17
CSE2	0.07	0.07
CSE3	0.11	0.10

## Conclusion

Here we make the first comprehensive genome-wide analysis of the CSP gene family in 22 mosquito species, identifying and naming 283 CSP genes. Characteristic comparisons and phylogenetic analyses of these 283 genes suggested that eight groups of genes (CSP1-8) are shared across almost all mosquito species. Within each CSP group, gene structure is similar and aa sequence identity is high. We thus propose that genes within a group are homologous and perform similar functions across different mosquito species. In the Culicinae species, three additional group genes (CSE1-3) were found, in much larger numbers than those in CSP1-8. Most of CSP genes in Culicinae were distributed in clusters on the scaffolds. We suggest that the CSP family of genes is highly conserved in mosquitos, and that they evolve slowly with purifying selection. This comparative genomic study on the CSPs of 22 mosquito species provides a comprehensive framework for further investigation of possible CSP functions.

## Supporting information

S1 Fig Alignment of groups of CSP genes (CSP1-8) in the 22 mosquito species analyzed. Conserved cysteines are represented by a red box. The alpha helical domains (α1-α6) identified in the chemosensory protein are marked by spiral line above the alignment. CSP5 is shown only up to the aa 187 as the C-terminal of this gene had long aa sequence extension.
(TIF)

S2 Fig Alignment of each group of CSP genes (CSE1-3) in the three Culicinae species. (TIF)

S1 File Detailed information and intron-exon organization of the CSP genes in the 22 mosquito species. (XLS)

S2 File Percent identity and coverage between amino acid sequences in each CSP group (CSP1-8, CSE1-3) of the 22 mosquito species. (XLS)

S3 File The average Ka/Ks ratio in each of the 22 mosquito species, as estimated by HyPhy and PAML. (XLS)
